# One-carbon metabolism biomarkers and genetic variants in relation to colorectal cancer risk by *KRAS* and *BRAF* mutation status

**DOI:** 10.1371/journal.pone.0196233

**Published:** 2018-04-25

**Authors:** Robin Myte, Björn Gylling, Jenny Häggström, Jörn Schneede, Anna Löfgren-Burström, Jeroen R. Huyghe, Göran Hallmans, Klaus Meyer, Ingegerd Johansson, Per Magne Ueland, Richard Palmqvist, Bethany Van Guelpen

**Affiliations:** 1 Department of Radiation Sciences, Oncology, Umeå University, Umeå, Sweden; 2 Department of Medical Biosciences, Pathology, Umeå University, Umeå, Sweden; 3 Department of Statistics, Umeå School of Business and Economics, Umeå University, Umeå, Sweden; 4 Department of Clinical Pharmacology, Pharmacology and Clinical Neurosciences, Umeå University, Umeå, Sweden; 5 Fred Hutchinson Cancer Research Center, Seattle, Washington, United States of America; 6 Department of Biobank Research, Public Health and Clinical Medicine, Umeå University, Umeå, Sweden; 7 Bevital AS, Bergen, Norway; 8 Department of Odontology, Cariology, Umeå University, Umeå, Sweden; 9 Department of Clinical Science, University of Bergen, Bergen, Norway; 10 Laboratory of Clinical Biochemistry, Haukeland University Hospital, Bergen, Norway; University of Crete, GREECE

## Abstract

Disturbances in one-carbon metabolism, intracellular reactions involved in nucleotide synthesis and methylation, likely increase the risk of colorectal cancer (CRC). However, results have been inconsistent. To explore whether this inconsistency could be explained by intertumoral heterogeneity, we evaluated a comprehensive panel of one-carbon metabolism biomarkers and some single nucleotide polymorphisms (SNPs) in relation to the risk of molecular subtypes of CRC defined by mutations in the *KRAS* and *BRAF* oncogenes. This nested case-control study included 488 CRC cases and 947 matched controls from two population-based cohorts in the Northern Sweden Health and Disease Study. We analyzed 14 biomarkers and 17 SNPs in prediagnostic blood and determined *KRAS* and *BRAF* mutation status in tumor tissue. In a multivariate network analysis, no variable displayed a strong association with the risk of specific CRC subtypes. A non-synonymous SNP in the *CTH* gene, rs1021737, had a stronger association compared with other variables. In subsequent univariate analyses, participants with variant rs1021737 genotype had a decreased risk of *KRAS*-mutated CRC (OR per allele = 0.72, 95% CI = 0.50, 1.05), and an increased risk of *BRAF*-mutated CRC (OR per allele = 1.56, 95% CI = 1.07, 2.30), with weak evidence for heterogeneity (P_heterogeneity_ = 0.01). This subtype-specific SNP association was not replicated in a case-case analysis of 533 CRC cases from The Cancer Genome Atlas (P = 0.85). In conclusion, we found no support for clear subtype-specific roles of one-carbon metabolism biomarkers and SNPs in CRC development, making differences in CRC molecular subtype distributions an unlikely explanation for the varying results on the role of one-carbon metabolism in CRC development across previous studies. Further investigation of the *CTH* gene in colorectal carcinogenesis with regards to *KRAS* and *BRAF* mutations or other molecular characteristics of the tumor may be warranted.

## Introduction

Disturbances in one-carbon metabolism, intracellular reactions involved in nucleotide synthesis and methylation and the target of antifolate chemotherapy, likely increase the risk of colorectal cancer (CRC) [1, 2]. The role of one-carbon metabolism in CRC development has been extensively investigated in observational studies [3], experimental studies [2, 4], and randomized clinical trials [5]. Although findings have shown some degree of inconsistency, balanced one-carbon metabolism is essential for genetic stability and may prevent tumor initiation, whereas imbalances may facilitate the progression of established tumors or precancerous lesions [2, 4].

CRC develops through distinct pathways resulting in molecular subtypes differing in clinical characteristics [6–8]. Two critical events in early CRC development are mutations in the *KRAS* and *BRAF* oncogenes [9]. Mutations in *KRAS* and *BRAF* are essentially mutually exclusive and occur in 30–50% and 4–18% of patients, respectively [10–12]. Clinically, mutated *KRAS* indicates resistance to anti-EGFR therapy and mutated *BRAF* is associated with an unfavorable prognosis [9]. Both mutations are more commonly found in women and proximal colon cancer [11]. Mutated *BRAF* is also more prevalent in older patients, in tumors originating from serrated lesions, tumors with extensive hypermethylation of CpG islands in tumor suppressor gene promoters (i.e., the CpG island methylator phenotype (CIMP)), as well as tumors with microsatellite instability (MSI) caused by malfunctioning DNA mismatch repair [6, 10].

CRC subtypes defined by *KRAS* and *BRAF* mutation status are largely representative of separate CRC developmental pathways with potential differences in etiology [6, 13]. For example, low plasma adiponectin levels are associated with *KRAS*-mutated, but not *KRAS* wild-type, CRC risk [14], and aspirin use is associated with *BRAF*-mutated, but not *BRAF* wild-type, CRC risk [15]. Similar differences for components of one-carbon metabolism and *KRAS* and *BRAF* mutations are biologically plausible [13, 16]. For instance, altered folate status is associated with aberrant global DNA methylation patterns [16]. which may primarily affect the progression of the hypermethylation-associated *BRAF-*mutated CRC subtype [6]. Yet, to our knowledge, no study has investigated one-carbon metabolism components in relation to risk of molecular subtypes of CRC defined by *KRAS* and *BRAF* mutation status.

To further scrutinize the role of one-carbon metabolism in CRC development, we investigated a comprehensive panel of 14 circulating biomarkers and 17 single nucleotide polymorphisms (SNPs) in relation to the risk of molecular subtypes of CRC defined by *KRAS* and *BRAF* mutation status in a case-control study nested within two population-based cohorts of the Northern Sweden Health and Disease Study.

## Methods

### Study population

This was a nested case-control study of two cohorts within the prospective, population-based Northern Sweden Health and Disease Study (NSHDS): the ongoing Västerbotten Intervention Programme (VIP, 78% of the participants, men and women) and the Mammography Screening Project in Västerbotten (MSP, 22% of the participants, all women), both described elsewhere [17]. At the final date for case identification for this study (March 31, 2009), the VIP included 83 621 participants and the MSP 28 802 participants.

### Study participants

**Fig 1:**
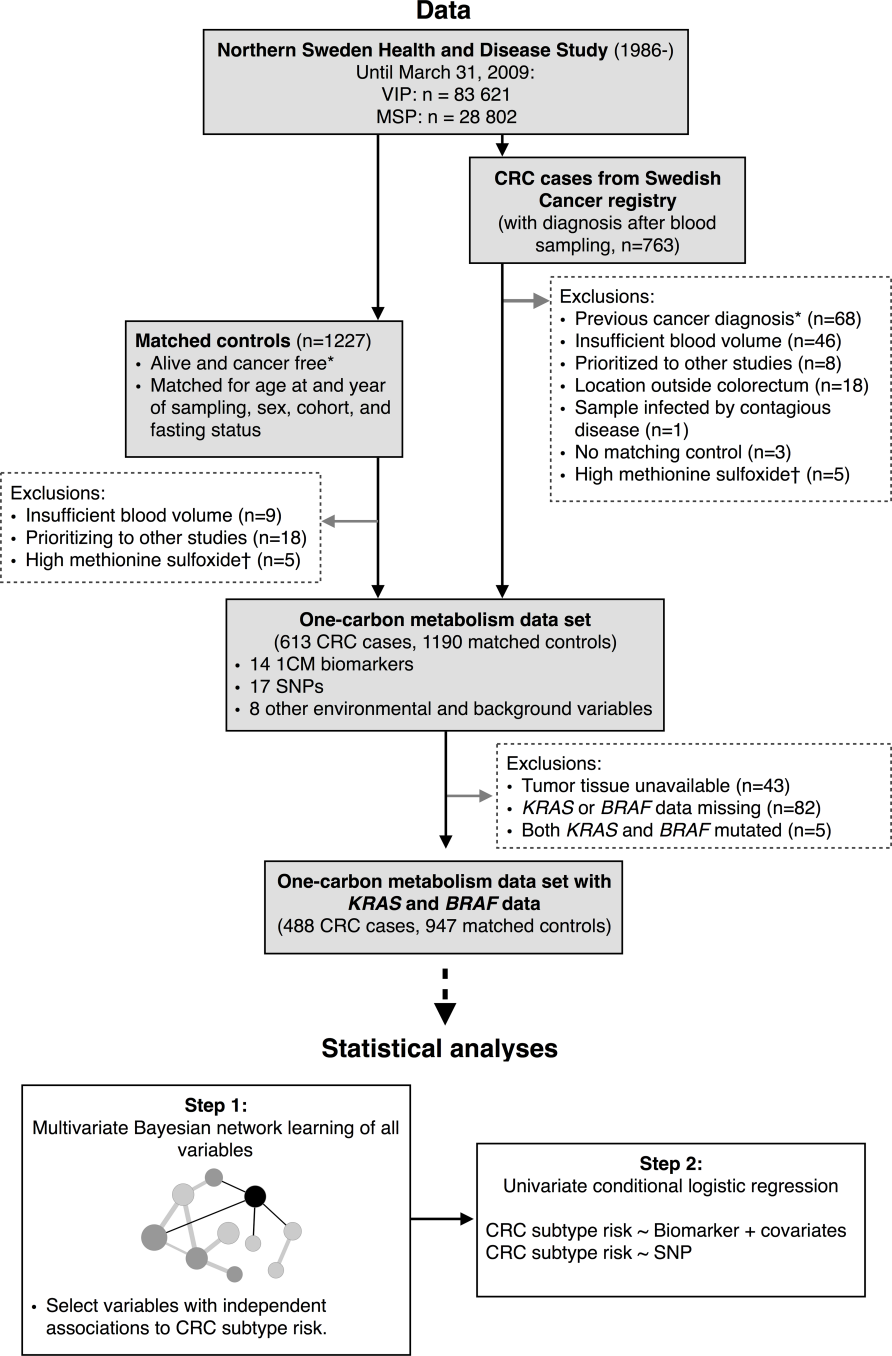
Study design. Illustrating the selection of participants based on the availability of one-carbon metabolism data in CRC cases and matched controls, and availability of *BRAF* and *KRAS* mutation status data in the CRC cases. *Other than non-melanoma skin cancer. †High methionine sulfoxide, indicates sample degradation. Abbreviations: CRC: Colorectal cancer, VIP: Västerbotten Intervention Programme, MSP: Mammography Screening Project.

CRC cases diagnosed between October 17, 1986, and March 31, 2009, with prediagnostic blood samples were identified by linkage with the Cancer Registry of Northern Sweden (ICD-10 C18.0 and C18.2–C18.9 for colon, C19.9 and C20.9 for rectum), with essentially complete inclusion. All cases and clinical data were verified by one gastrointestinal pathologist. For each case, two controls were randomly selected, matched by sex, age at and year of blood sampling and data collection, fasting status, and cohort (VIP or MSP). A total of 613 cases and 1190 controls were previously analyzed for the biomarker/metabolite panel in relation to overall CRC risk [18]. This list was used to acquire archival tumor tissue from the CRC cases. After exclusions, the final dataset included 488 cases and 947 controls with both available one-carbon metabolism and *KRAS* and *BRAF* mutation status data (**Fig 1**). The study was approved by the ethical committee at Umeå University (Regional Ethical Review Board in Umeå, Sweden, 03–186). All participants gave a written informed consent.

### Blood analyses

Venous blood samples in the NSDHS were aliquoted and EDTA plasma was frozen at -80°C within one hour of collection, or at -20°C for at most one week prior to storage at -80°C. In the VIP cohort, plasma samples were collected in the morning, and a majority (77%) had fasted more than 8 hours prior to sampling. In the MSP cohort, sampling was spread out during the day, and most participants (96%) had fasted less than 4 hours. All biochemical analyses were performed at Bevital AS (http://www.bevital.no/, Bergen, Norway). The biomarkers were measured in EDTA plasma using liquid or gas chromatography–mass spectrometry or microbiological methods as previously described [18]. Between-day coefficients of variation (CV) varied between 2–13% [19, 20]. SNPs were determined using MALDI-TOF mass spectrometry [21]. The biomarkers were chosen based on previous studies of one-carbon metabolism and CRC risk [3], and to capture a wide array of aspects of one-carbon metabolism while maintaining an adequate marker stability and reproducibility [19, 20]. To adjust biomarker associations for genetic confounders and assess associations of genetically determined one-carbon metabolism function, we also included a panel of SNPs in genes involved in one-carbon metabolism, some of which had previously been studied in relation to CRC [22]. In total, 31 exposures were investigated, namely 14 plasma biomarkers: folate, vitamin B6 (pyridoxal 5'-phosphate, PLP), vitamin B2 (riboflavin), vitamin B12 (cobalamin), homocysteine, cystathionine, cysteine, glycine, serine, methionine, choline, betaine, dimethylglycine (DMG), and sarcosine, and 17 SNPs in 13 genes: *BHMT (*rs3733890), *CBS (*rs234706, 844ins68), *CTH* (rs1021737), *DHFR* (rs70991108), *FOLR1* (rs2071010), *MTHFD1* (rs2236225), *MTHFR* (rs1801131, rs1801133), *MTR* (rs1805087), *MTRR* (rs1532268, rs1801394), *SHMT* (rs1979277), SLC19A1 (rs1051266), *TCN2* (rs1801198, rs9606756), and *TYMS* (rs34489327) (see **Table A** in **S1 File** for a detailed information on all SNPs).

### Tumor tissue analyses

In CRC cases, DNA was extracted and purified from formalin-fixed, paraffin-embedded tumor tissue collected during routine clinical practice at the Department of Clinical Pathology, Umeå University Hospital, Umeå, Sweden. *KRAS* was analyzed by sequencing the activating mutations in codon 12 and 13 using Big Dye v. 3.1, according to the manufacture protocol (Applied Biosystems, Life Technologies, Foster City, CA, USA) [23]. The mutational status of BRAF^V600E^ was analyzed using TaqMan allelic discrimination assay (reagents from Applied Biosystems) and digital droplet PCR (reagents from Bio-Rad Laboratories, Hercules, CA, USA) [24]. CIMP status was determined using MethyLight real-time PCR and an 8-gene panel as previously described in [25]. Cases were classified as CIMP-negative if there was no promotor methylation in any of the eight genes defined as a percentage of methylated reference (PMR) value above 10%; CIMP-low if there was promotor methylation in one to five genes; and CIMP-high if there was promotor methylation in six to eight genes. MSI status was assessed by immunohistochemical analysis of MLH1, MSH2, MSH6, and PMS2 [25]. Samples lacking tumor cells with nuclear staining for any of the mismatch repair proteins were categorized as MSI.

### Variables

CRC cases were classified as *KRAS*-mutated, *BRAF*-mutated, or *KRAS*/*BRAF* wild-type. Missing values for the biomarkers and SNPs were assumed to be missing at random and were omitted separately for each analysis (0–3% missing per variable). Other variables included in the multivariate or multivariable models were age at and year of blood sampling (divided into quartile groups), sex (male, female), fasting status (<4, 4–8, ≥8 hours), cohort (VIP, MSP), smoking status (never, current, ex-smoker), body mass index (BMI, <25, 25–30, ≥30 kg/m^2^), estimated glomerular filtration rate (eGFR) as a marker for kidney function calculated by the Cockcroft-Gault formula (based on plasma creatinine levels, age, sex and body weight, in tertile groups, with cut-offs based on the control participant distribution), and plasma neopterin concentrations, a marker of immune system activation (tertiles) [26]. For the VIP cohort, we also had self-reported information on alcohol intake (zero intake or above/below sex-specific medians), recreational physical activity (5-level scale from never to >3 times/week) and occupational physical activity (5-level scale from sedentary to physically strenuous most of the time). Missing values for these variables were assigned to a separate “missing” category.

### Statistical analysis

The primary statistical analysis was conducted in two steps (see **Fig 1**). First, to account for complex interrelations between biomarkers, SNPs, and lifestyle variables, we modeled all variables in relation to CRC risk by *KRAS* and *BRAF* mutation status simultaneously in multivariate Bayesian networks. Bayesian network analysis uses the data to estimate independent associations between all variables, both exposures and outcomes, and presents these as a network. As all associations are estimated simultaneously, the total number of analyses is minimized, reducing the risk of chance findings due to multiple testing. This approach has previously been applied for similar purposes [18, 27, 28]. The Bayesian networks were estimated with machine learning using the bnlearn R-package [29, 30]. Briefly, in 1000 bootstrap samples, networks were estimated on discrete data with the Hill-climbing (HC) algorithm using the Akaike information criterion (AIC) score. The continuous biomarkers were included as discretized tertile variables, using cut-offs based on the distribution of the controls. SNPs were included as dichotomous variables (common or variant genotype). The final networks were obtained by averaging over the 1000 bootstrap networks. An edge (i.e., independent association) between two variables was included if its edge confidence, defined as the proportion of times an edge was present among the 1000 bootstrap networks, was above an estimated significance threshold [30]. Edge confidence was also used to measure strength of the association to CRC subtype risk for each one-carbon metabolism variable. The multivariate analysis was based on participants with complete case data on biomarkers and SNPs (446 cases and 867 controls, after excluding 42 cases and 80 controls with any missing biomarker or SNP data).

Variables with the strongest associations with CRC subtype risk in step 1 were considered for multivariable, univariate analysis in step 2. In the univariate analyses, we estimated subtype-specific odds ratios (ORs) per 1 SD increase in biomarker levels or per allele of the SNPs by conditional logistic regression. Heterogeneity of the risk associations by *BRAF* and *KRAS* mutation status were tested with likelihood ratio tests, comparing a model in which the risk association could vary across subtypes to a model in which all associations were held constant across subtypes [31]. Biomarker estimates were adjusted for the matching variables by conditioning the model on the matched case-control sets, and BMI, smoking status, alcohol intake, plasma neopterin, and recreational and occupational physical activity by regression. SNP estimates were only adjusted for the matching variables by conditioning. All biomarkers and all SNPs had similar associations to overall CRC risk in the VIP and MSP cohorts (Cochran’s Q tests P > 0.05). Therefore, the primary analysis was conducted on combined data from VIP and MSP to maximize power.

To account for potential selection bias from unavailable *KRAS* and *BRAF* mutation data in some CRC cases, we conducted a sensitivity analysis by estimating subtype-specific associations using inverse probability weighted (IPW) conditional logistic regression models [32]. In short, we first fitted a logistic regression model in all cases with tumor data availability as the outcome and potential predictors of data availability as covariates. Included covariates were tumor stage, tumor site, age at diagnosis, year of diagnosis (<2002, 2002–2006, ≥2006), cohort, and sex. Cases without data on tumor stage or site were excluded from these analyses (n = 29). Fitted probabilities, *p_i_*, were then estimated using the model for each case *i*. Finally, conditional logistic regression models for CRC subtype risk by all variables were fitted as in the complete case analysis, but with weights set to 1 for controls, $\frac{1}{p_{i}}$ for cases with available tumor data, and 0 for cases without available tumor data (i.e., not included in the estimation). Estimates from these models are adjusted for selection bias potentially caused by the observed covariates.

To externally validate the potential SNP finding, we utilized The Cancer Genome Atlas (TCGA) data generated by the TCGA Research network: https://cancergenome.nih.gov/. Specifically, case-case analyses were made for 533 colorectal cancer patients with data available for both somatic mutation calls and germline Affymetrix Genome-Wide Human SNP6.0 array data that passed our QC as described below. *KRAS* (activating mutations in codon 12 or 13) and *BRAF* (*BRAF*^V600E^) mutation status for TCGA COAD + READ individuals was obtained by querying NCI's Genomic Data Commons (GDC) repository (https://gdc.cancer.gov). Germline SNP genotypes were obtained by imputation to the Haplotype Reference Consortium. In brief, we processed raw Affymetrix Genome-Wide Human SNP 6.0 array data using a standardized GWAS QC pipeline. Genotypes called with the Birdseed genotype-calling algorithm were set to missing if their confidence score was >0.1. Samples with genotype missing call rate (<98%), and samples with mismatches between genotypic and reported sex were excluded. SNPs were filtered based on call rate (<98%), and Hardy-Weinberg Equilibrium (HWE) test P-value (<0.0001). We estimated haplotype phase using SHAPEITv2 [33] and imputed to the Haplotype Reference Consortium (HRC) panel [34] using the SNP6 array data as imputation target. Ratio of odds ratios (RORs) per allele of the SNPs were calculated for *KRAS*-mutated and *BRAF*-mutated tumors vs *KRAS*/*BRAF* wild type tumors using multinomial logistic regression. Heterogeneity in the SNP-CRC *KRAS/BRAF* subtype association was tested with a likelihood ratio test. For comparison, identical case-case analyses were made for the NSHDS.

All tests were 2-sided when applicable, and P-values < 0.05 were considered statistically significant. All computations were conducted in R v.3.4.2 (R Foundation for Statistical Computing, Vienna, Austria).

## Results

### Baseline and case characteristics

A total of 125 of the 488 cases (26%) were *KRAS* mutated and 117 cases (24%) were *BRAF* mutated (**Table 1**). *BRAF-*mutated cases were characterized by a higher proportion of females, tumors located in the right-sided colon, CIMP-high status tumors, and MSI tumors, as well as slightly higher BMI and lower alcohol intake at baseline, compared to the other subtypes. *KRAS*/*BRAF* wild-type cases were characterized by a lower age at diagnosis and a higher proportion of rectal tumors. Median plasma concentrations and means and standard deviations of log-plasma concentrations of the one-carbon metabolism biomarkers are presented in **Table B** in **S1 File**.

**Table 1:** Baseline and clinical characteristics of colorectal cancer cases by *KRAS* and *BRAF* mutation status and matched controls.

	Controls (n = 947)	Cases^a^ (n = 488)		
Variable		*KRAS*-mutated (n = 125)	*BRAF*-mutated (n = 117)	*KRAS/BRAF* wild-type (n = 246)
**Cohort (%)**				
VIP	739 (78)	107 (86)	85 (73)	188 (76)
MSP	208 (22)	18 (14)	32 (27)	58 (24)
**Sex (%)**				
Male	375 (40)	49 (39)	31 (26)	115 (47)
Female	572 (60)	76 (61)	86 (74)	131 (53)
**Age (years)**^**b**^	59.8 (50.2–60.2)	59.8 (49.9–60.1)	59.8 (50.8–60.2)	59.8 (50.2–60.1)
**Body mass index (BMI, kg/m**^**2**^**)**^**b**^	25.7 (23.4–28.1)	25.3 (22.7–27.5)	26.3 (23.7–28.6)	25.9 (23.8–28.4)
**Smoking status (%)**				
Never smoker	607 (64)	73 (58)	67 (57)	149 (61)
Current smoker	191 (20)	23 (18)	27 (23)	42 (17)
Ex-smoker	149 (16)	29 (23)	23 (20)	55 (22)
**Alcohol intake (g/day)**^**b**^^,^^**c**^	2.3 (0.2–5.3)	2.4 (0.2–5.7)	0.7 (0.1–4.6)	2.7 (0.5–6.5)
**Recreational physical activity**^**c**^^,^^**d**^^,^				
1	272 (38)	53 (50)	40 (49)	82 (45)
2	192 (27)	25 (24)	22 (27)	40 (22)
3	136 (19)	16 (15)	12 (15)	33 (18)
4	59 (8)	6 (6)	3 (4)	15 (8)
5	50 (7)	5 (5)	5 (6)	14 (8)
**Occupational physical activity**^**c**^^,^^**e**^ **(%)**				
1	126 (20)	20 (23)	11 (16)	37 (22)
2	120 (19)	11 (12)	17 (25)	24 (14)
3	184 (29)	22 (25)	17 (25)	52 (31)
4	172 (27)	29 (33)	18 (27)	33 (20)
5	38 (6)	6 (7)	4 (6)	20 (12)
**Plasma neopterin** (nmol/L)^**b**^	9.4 (8.0–11.4)	9.6 (7.8–11.3)	9.8 (8.3–11.9)	9.5 (8.0–11.9)
**Age at diagnosis (years)**^**b**^		65.8 (58.0–71.8)	67.2 (62.7–72.1)	64.2 (58.3–69.2)
**Follow-up time (years)**^**b**^		9.4 (5.1–13.6)	8.7 (5.2–12.2)	7.3 (4.2–10.4)
**Site (%)**^**f**^				
Right-sided colon		40 (32)	70 (60)	47 (19)
Left-sided colon		41 (33)	35 (30)	90 (37)
Rectum		44 (35)	12 (10)	108 (44)
**Stage (%)**^**f**^				
I&II		69 (59)	54 (47)	134 (56)
III&IV		47 (41)	60 (53)	107 (44)
**CIMP status (%)**^**f**^				
Negative		54 (55)	9 (10)	127 (63)
Low		42 (43)	31 (33)	63 (32)
High		2 (2)	54 (57)	10 (5)
**MSI status (%)**^**f**^				
MSI		2 (2)	46 (48)	18 (9)
MSS		97 (98)	49 (52)	185 (91)

IQR: interquartile range, CIMP: CpG Island methylator phenotype, MSI: Microsatellite instability, MSS: Microsatellite stable

a 5 cases with mutations in both *KRAS* and *BRAF* (1% of cases), and their corresponding controls, were excluded from the analyses.

b Median and interquartile range (IQR), 25-75^th^ percentile.

c Variables only available for the VIP cohort.

d Self-reported exercise frequency during leisure time on a scale from 1–5, where 1 = never, 2 = every now and then—not regularly, 3 = 1–2 times/week, 4 = 2–3 times/week, 5 = more than 3 times/week.

e Self-reported on a scale from 1–5, where 1 = sedentary or standing work, 2 = light but partly physically active, 3 = light and physically active, 4 = sometimes physically straining, 5 = physically straining most of the time.

f Site could not be determined for 1 case, stage could not be determined for 29 cases, CIMP status could not be determined for 96 cases, and MSI status could not be determined for 91 cases.

### Multivariate analysis using Bayesian network learning

**Fig 2:**
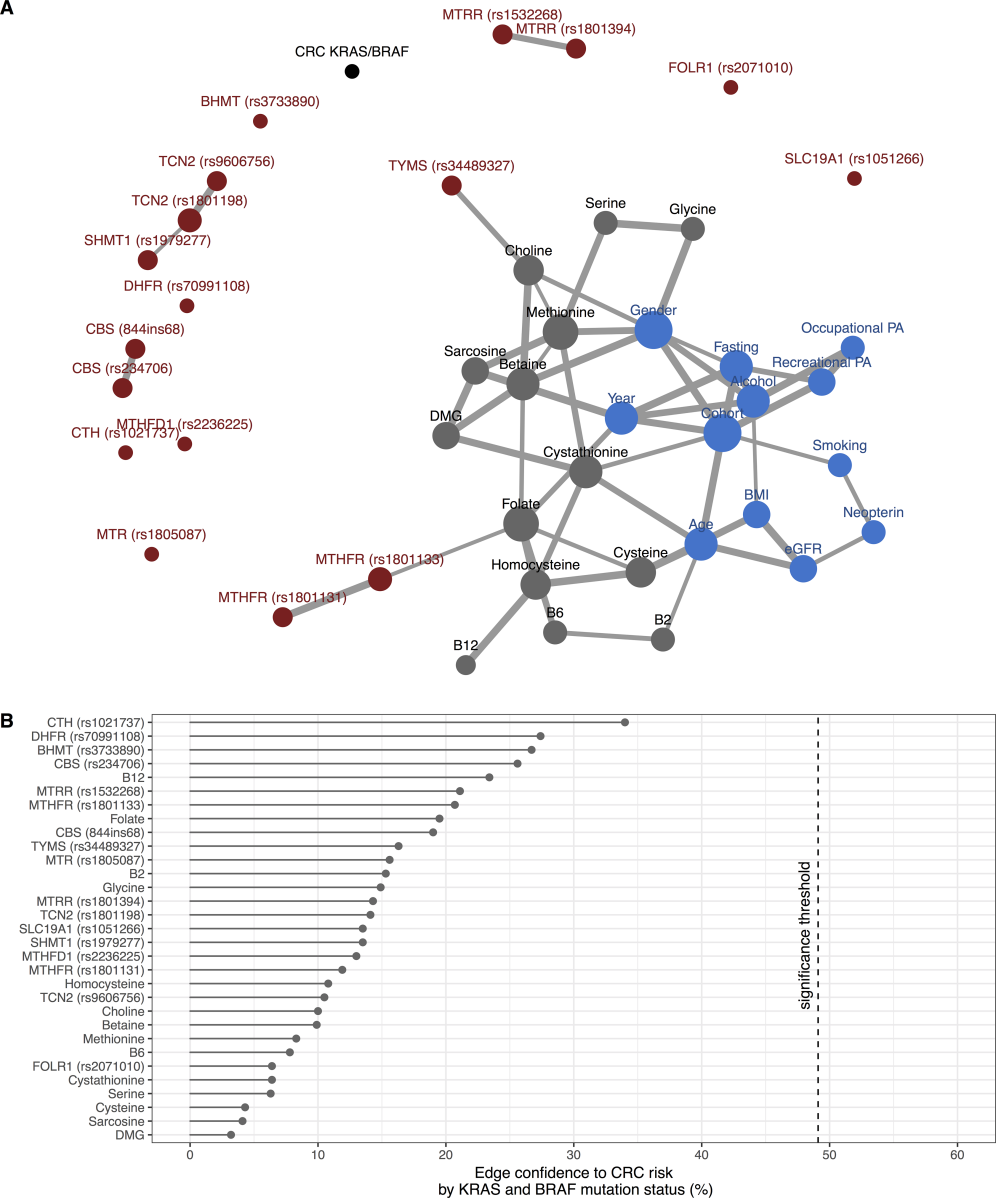
Multivariate Bayesian network learning results. **(A)** Bayesian network of all included variables estimated from data with the Hill-climbing (HC) algorithm, averaged over 1000 bootstrap replicates. An edge between two variables indicates an association independent of all other variables in the network. Edge thickness corresponds to the strength of association, measured by edge confidence (proportion of times an edge was present in 1000 bootstrap sample networks). Node size corresponds to the number of edge connections (i.e., number of independent associations with other variables) **(B)** Strength of association to CRC risk by *KRAS* and *BRAF* mutation status for each biomarker and SNP. Abbreviations: BMI: Body mass index, CRC: Colorectal cancer, DMG: Dimethylglycine, eGFR: estimated glomerular filtration rate, PA: Physical activity.

Independent associations between all one-carbon metabolism biomarkers, SNPs, and lifestyle or background variables, estimated using Bayesian network analysis, is presented as a network in **Fig 2A**. An edge between two variables indicates an association independent of all other variables in the network. Associations between variables in the network largely corresponded to expected biological relationships. **Fig 2B** shows association strengths for each investigated one-carbon metabolism variable in relation to CRC risk by *KRAS* and *BRAF* mutation status measured by edge confidence, defined as the proportion of times an independent association was present in the 1000 bootstrap networks. No variable exhibited a significant independent association to subtype-specific CRC risk in this multivariate analysis (i.e., edge confidence above the estimated significance threshold of 49%). The variable with the top edge confidence, i.e., the variable with a slightly stronger association to CRC subtype risk compared to other variables, was a SNP in the *CTH* gene, rs1021737 (edge confidence: 34%).

### Univariate analyses

The *CTH* rs1021737 SNP, which displayed a stronger association with subtype-specific CRC risk in the Bayesian network analysis compared to all other variables, was carried on for univariate testing. ORs for CRC risk by *KRAS* and *BRAF* mutation status per allele of the *CTH* rs1021737 SNP, estimated by univariate conditional logistic regression and adjusted for potential confounders, are presented in **Table 2**. Participants with the variant *CTH* rs1021737 genotype had a decreased risk of *KRAS*-mutated CRC (OR per allele = 0.72, 95% CI = 0.50, 1.05), and an increased risk of *BRAF*-mutated CRC (OR = 1.56, 95% CI = 1.07, 2.30), but with weak evidence for heterogeneity (P_heterogeneity_ = 0.01). This would not be statistically significant if a conservative, Bonferroni-adjusted significance threshold of 0.002 (0.05/31 ≈ 0.002) for sequential univariate testing of all exposure variables was applied. Results were similar between the VIP and MSP cohorts, men and women, colon and rectal cancer, as well as when removing cases with follow-up time between sampling and diagnosis below 2 years, or removing participants with low plasma folate levels (<6 nmol/L) (**Table C** in **S1 File**). Complete univariate results for all one-carbon metabolism variables are presented in **Table D** in **S1 File**. Two variables other than the rs1021737 SNP also displayed nominally significant heterogeneity in the univariate analyses, vitamin B2 (P_heterogeneity_ = 0.02) and the rs3733890 SNP (P_heterogeneity_ = 0.03). However, the edge confidence in the multivariate analysis for these variables were low (15.3 and 26.7%, respectively), suggesting that these associations were either confounded or mediated by other factors, or chance findings.

**Table 2:** Odds ratios for CRC risk by *KRAS* and *BRAF* mutation status per allele of the *CTH* rs1021737 SNP.

Exposure	CRC subtype	Cases/controls (n)	OR (95% CI)^a^	P_heterogeneity_^b^
rs1021737 (per allele)	*KRAS*-mutated	124/239	0.72 (0.50, 1.05)	0.01
	*BRAF*-mutated	115/221	1.56 (1.07, 2.30)	
	*KRAS/BRAF* wild type	242/452	0.94 (0.72, 1.24)	

a Estimates from conditional logistic regression models adjusted for the matching variables: age at and year of blood sampling, cohort, fasting status, and sex.

b Likelihood ratio test (2-sided) of heterogeneity, testing for a common association across subtypes.

Associations between the *CTH* rs1021737 SNP and metabolites in the transsulfuration pathway are displayed in **Figure A** in **S2 File**. Although not evident in the estimated Bayesian network, median plasma cystathionine levels were approximately 7% higher per variant allele of the rs1021737 (P = 0.0009). No other biomarker was associated with the SNP. The associations were consistent in cases and controls.

### Sensitivity analyses

To evaluate potential selection bias based on tumor *KRAS*/*BRAF* mutation data availability, we first compared results for cases with and without available data. We saw no major differences in baseline and clinical characteristics (**Table E** in **S1 File)**. There was a slightly lower age at sampling and proportion of right-sided tumors for cases without available data (P = 0.04 and P = 0.03, respectively). Overall, there were no major differences in the associations between biomarkers or SNPs and overall CRC risk by tumor data availability (**Table F** in **S1 File**). Two variables (plasma glycine and the *CBS* 844ins68 SNPs) appeared only associated with CRC risk for cases without available tumor data (P_heterogeneity_ = 0.02 and 0.03). However, as these variables were not associated with overall CRC risk in the full data set and no other biomarkers or SNPs displayed any differences by tumor data availability, those heterogeneities by tumor data availability may be chance findings.

**Fig 3:**
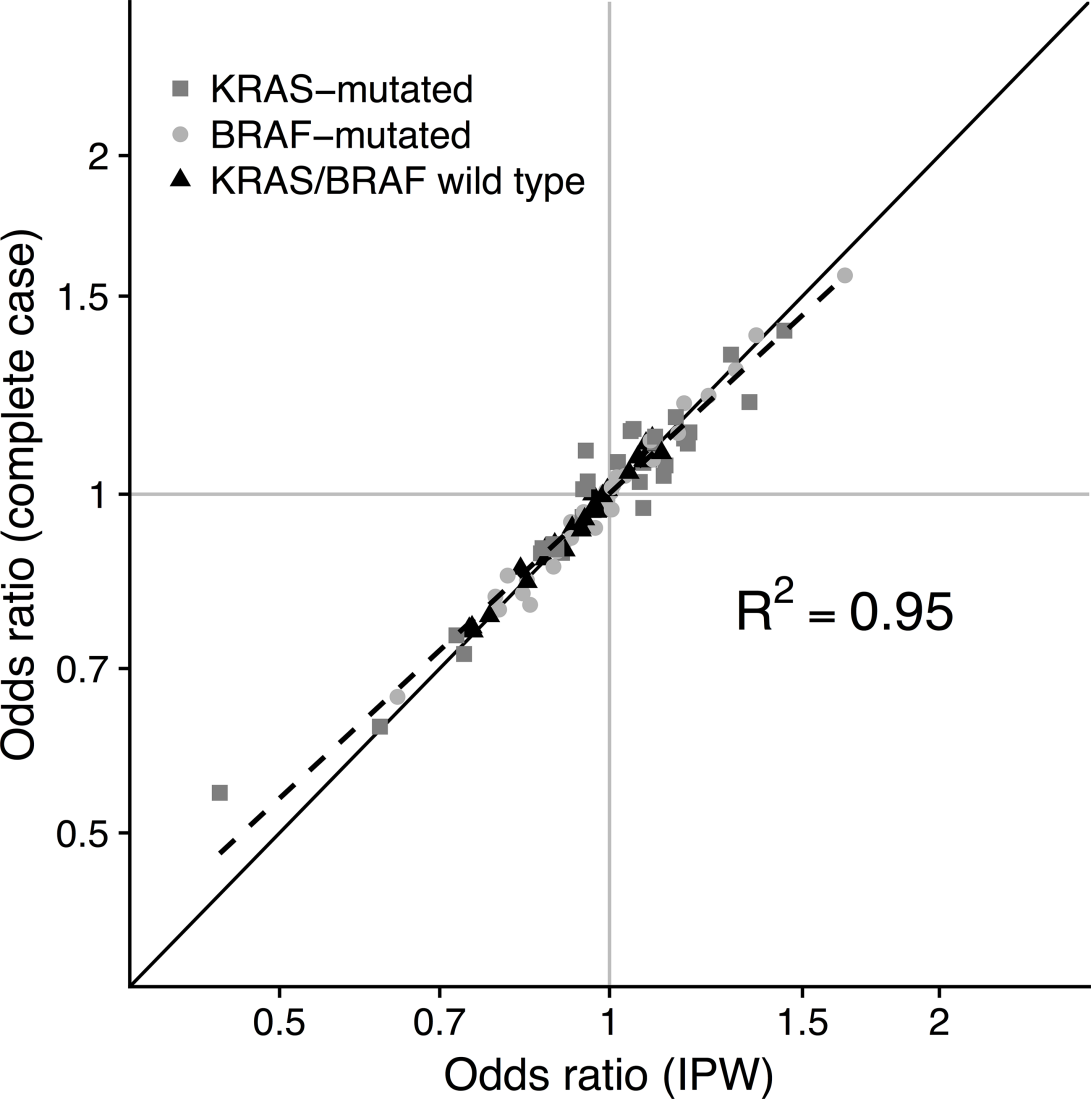
Comparison between odds ratios (ORs) for CRC risk by *KRAS*/*BRAF* mutation status for all one-carbon metabolism variables using complete-case models and selection-bias-adjusted IPW models. The 45-degree line represents values for which the complete case ORs are equal to the IPW ORs. The broken line represents a fitted regression line.

We also estimated ORs for CRC risk by *KRAS* and *BRAF* mutation status by all variables adjusted for potential selection bias using IPW conditional logistic regression, weighted by the estimated inverse probability of tumor *KRAS*/*BRAF* mutation data availability. We checked the ability of the data availability model to create balance in the predictors by refitting the model weighted by the inverse fitted probabilities from the first model ($\frac{1}{p_{i}}$ for cases with data, $\frac{1}{{1-p}_{i}}$ without data). Age at diagnosis, year of diagnosis, and tumor site were associated with tumor data availability in the first model (**Table G** in **S1 File**), but were null in the weighted model (all coefficients close to zero, P>0.2), indicating that covariate balance was achieved. In the IPW-weighted conditional logistic regression models for CRC risk by *KRAS* and *BRAF* mutation status, ORs were very similar to the complete case analysis, with no sign of a large systematic difference in ORs (**Fig 3**). The same was true for the log(OR) standard errors (data not shown).

To investigate if one-carbon metabolism biomarkers and SNPs had different association with CRC subtypes defined by CIMP or MSI status of the tumor, we repeated the multivariate and univariate analyses for these traits. Similar to the results for the CRC subtypes by *KRAS* and *BRAF* mutation status, no variable had a strong association with CRC subtype risk by CIMP or MSI status in Bayesian network models (**Figure B** in **S2 File**). In univariate models, no variable, including folate or the variables with the highest edge confidence to CRC subtype risk by CIMP or MSI status in the Bayesian network models, demonstrated differences in risk estimates in univariate models (**Tables H-I** in **S1 File**). Results were the same when participants from our previous study (190 cases, 380 controls [25]) were excluded (data not shown).

### TCGA replication of rs1021737 results

Case-case analyses of rs1021737 using the NSHDS data yielded a similar association to CRC risk by *KRAS* and *BRAF* mutation status as the case-control analyses, i.e., CRC cases with variant rs1021737 genotype were more likely to be *KRAS*-mutant, and less likely to be *BRAF*-mutant compared to *KRAS*/*BRAF* wild type (P = 0.04, Table 3). In TCGA cases, however, neither the associations nor heterogeneity in associations were replicated (P = 0.85).

**Table 3:** Replication of the *CTH* rs1021737 SNPs associations in The Cancer Genome Atlas (TCGA).

	NSHDS (n = 420)			Replication TCGA (n = 553)		
	n	ROR (95% CI)^a^	P^b^	n	ROR (95% CI)^a^	P^b^
*KRAS*/*BRAF* wild-type	242	1 (ref)	0.04	337	1 (ref)	0.85
*KRAS*-mutated	124	0.76 (0.52, 1.11)		169	1.01 (0.76–1.35)	
*BRAF*-mutated	115	1.33 (0.93, 1.89)		47	0.87 (0.54–1.43)	** **

**NSHDS:** Northern Sweden Health and Disease Study. **TCGA**: The Cancer Genome Atlas. **ROR**: Ratio of odds ratio, **CI**: Confidence interval.

**a** RORs per allele calculated in multinomial logistic regression models.

**b** Likelihood ratio test (2-sided) of heterogeneity, testing for a common association across subtypes.

## Discussion

This nested case-control study combined prospective blood samples and archival tumor tissue analyses from 488 colorectal cancer patients. A comprehensive panel of plasma biomarkers and SNPs involved in one-carbon metabolism was subjected to multivariate analysis using Bayesian network learning. We found no evidence of any clear subtype-specific associations between one-carbon metabolism variables and CRC risk by *KRAS* and *BRAF* mutation (or CIMP or MSI) status of the tumor. A non-synonymous SNP in the *CTH* gene, rs1021737, had a slightly stronger association to CRC subtype risk in the network models compared to other variables, with diametrically opposed relationship to CRC risk depending on *KRAS* and *BRAF* mutation status in univariate analyses and weak evidence for heterogeneity. The association was not replicated in case-case analysis in the TCGA.

Low plasma folate concentrations are associated with a decreased CRC risk in the NSHDS [35, 36], a consistent, somewhat unexpected observation that fueled our interest in possible etiological differences between molecular subtypes. In a smaller study from the NSHDS, the association for folate seemed possibly to be limited to CIMP-low/high tumors [25]. Heterogeneity by CIMP-status was not observed in the current study. These and other findings, including inverse associations between metabolites other than folate and CRC risk in the NSHDS [17], may support nucleotide synthesis, rather than DNA methylation, as the pathogenic mechanism. In the current study, we did not observe any clear differences in association between low folate and decreased CRC risk association across CRC subtypes defined by *KRAS* and *BRAF* mutation status. Lower folate levels may therefore hinder the progression of established tumors or precancerous lesions regardless of molecular subtype.

A potentially general harmful effect of high folate levels in the presence of precancerous lesions acting across tumor subtypes might fuel concerns over the safety of food folic acid fortification. However, CRC incidence in the United States decreased over time despite the implementation of mandatory folic acid fortification of flour and cereal [37]. If higher folate levels prevent tumor initiation in normal tissue but facilitate the progression of precancerous lesions, then the combination of folic acid fortification with extensive colonoscopy screening, allowing removal of precancerous lesions, may have contributed to the overall reduction in CRC incidence in the US [38]. This hypothesis is also in line with the lack of decrease in CRC incidence rates in Canada [37], where mandatory folic acid fortification was introduced at nearly the same time as the United States but organized screening programs do not include colonoscopy [39].

The *CTH* gene encodes for enzymes involved in the homocysteine transsulfuration pathway [2]. In our study, cystathionine concentrations increased with increasing numbers of variant T alleles of *CTH* rs1021737. The SNPs has been associated with a slight increase in homocysteine levels in two smaller studies [40], though not apparent in the present, larger study. The rs1021737 variant T allele may therefore be associated with decreased CTH enzyme activity. The SNP was not associated with overall CRC risk in the NSHDS [18]. No other study has, to our knowledge, investigated this SNPs in relation to CRC risk. Plasma homocysteine, cystathionine, and cysteine levels were unrelated to CRC or CRC subtype in the present and previous studies [35, 36, 41–44]. Our observation of opposite CRC risk associations for the rs1021737 by *KRAS* and *BRAF* mutations status in the NSHDS was not replicated in case-case analysis in the TCGA. The present study, therefore, provides no conclusive evidence for a role of the SNP in *KRAS* or *BRAF*-mutated colorectal cancer development. However, experimental studies are providing increasing support for a role of the two main transsulfuration pathway enzymes CBS and CTH in colorectal cancer development through production of the gasotransmitter hydrogen sulfide [45–48]. If *CTH* rs1021737 variant carriers do have decreased enzyme activity, then a role for H_2_S in preventing *KRAS*-mutated CRC and promoting *BRAF*-mutated CRC would be consistent with our observations for the rs1021737 SNP in the NSHDS. Additional studies of SNPs in the *CBS* and *CTH* genes and the risk of CRC or CRC subtypes might, therefore, be warranted.

The 17 SNPs included in the study were originally selected for investigating gene-environmental interactions, especially the importance of B-vitamin status on SNP functional impact. Therefore, the panel do not capture the entire genetic contribution to one-carbon metabolism or tag the individual genes. Yet, most included SNPs were non-synonymous, and one SNP, the extensively studied *MTHFR* rs1801133 (often referred to as *MTHFR* 677C>T), is a functional SNP known to reduce enzyme activity [49]. Despite considerable heritability in circulating one-carbon metabolites (e.g., twin-based heritability for folate levels: 56% [50]) and plentiful evidence for a role of one-carbon metabolism in CRC development [1, 2], SNPs in one-carbon metabolism-related genes are not strong predictors in genome-wide association studies (GWAS) of CRC [51]. If there is no link between SNPs in these genes and overall CRC risk, heterogeneity among associations with CRC subtypes would have to be strongly opposite to mask an overall association. Our null results for the SNPs may, therefore, be expected. However, such opposite associations with CRC subtypes are biologically plausible given the large differences in molecular and pathological features among tumors [13].

The *BRAF* mutation frequency of 24% in our study is higher than the typically observed range of 4–18% [12]. Another Swedish population-based study of metastatic CRC tumors observed a similarly high *BRAF* mutation frequency of 21%, likely caused by the unselected study population [52]. The same explanation might hold true for our study in the NSHDS, given the high participation rate and low selection bias in the large VIP cohort [53, 54]. Furthermore, the use of the Swedish Cancer Registry for CRC diagnosis follow-up, which is essentially complete due to mandatory reporting by law, minimizes the risk of a selected CRC case population [55]. The *BRAF* mutation frequency in our less-selected study population may, therefore, more closely reflect the true mutation frequency in the population. The TCGA participants, on the other hand, originates from a North American population and has lower a *BRAF* mutation frequency (9% of TCGA participants in this study) and likely a higher folate status (caused by the mandatory folic acid fortification of flour and cereal and a higher supplemental vitamin intake) compared to the NSHDS. This may affect the comparability between the NSHDS and the TCGA, and could be an alternative explanation to the inconsistent *CTH* rs1021737 results.

There are some limitations to our study. *KRAS* and *BRAF* data were unavailable for a portion of the CRC cases. However, we saw no indications of a strong selection, associations to overall CRC risk did not differ by the availability of *KRAS*/*BRAF* data in the cohort, and IPW analyses taking selection bias caused by observed characteristics such as tumor stage, tumor site, and age at and year of diagnosis into account did not alter the results. Another limitation was that only *KRAS* codons 12 and 13, those routinely analyzed in clinical practice at the time, were assessed. Although these codons cover the bulk of *KRAS* mutations, we observed lower frequencies of *KRAS* mutations than could be expected from a more extensive analysis. Also, NRAS mutations were not assessed. A single prediagnostic blood sample from each participant was analyzed, but issues of storage stability and within-individual reproducibility of the biomarkers have previously been addressed [19, 20], and are unlikely to have any material impact on the results. Rather than the limited SNP panel available for this study, detailed genetic data, using a GWAS approach for example, would have allowed more comprehensive analyses of genetically determined one-carbon metabolism in relation to CRC subtypes. Lastly, using a limited set of molecular markers to classify CRC into subtypes cannot completely capture intertumoral heterogeneity [8], which could cause interpretive bias if the subtypes assessed does not represent pathogenic mechanisms [56]. Yet, the fact that the mutually exclusive mutations in *KRAS* and *BRAF* are early events in the carcinogenic process [9], and the recent evidence for subtype-specific risk associations for seemingly unrelated factors [14, 15], both support the notion of homogenous pathogenic mechanisms within subtypes defined by *KRAS* and *BRAF* mutation status.

The main strengths of the study include the prediagnostic blood samples (of high quality with regard to collection, handling, and storage) from the large population-based NSHDS, in combination with postdiagnostic clinical and molecular tumor data. This type of molecular pathological epidemiology approach is important for investigations of CRC etiology, given the expectedly large intertumoral heterogeneity [7, 13]. The nested case-control design was also a strength of our study compared to the often-used case-case design, which can assess heterogeneity of risk associations between subtypes but cannot estimate the magnitude or direction of risk associations for each subtype. We also used a comprehensive panel of biomarkers, as well as a few SNPs, related to one-carbon metabolism and accounted for several potential lifestyle-related confounders. The study population is generally characterized by low folate status [57], allowing the study of risk relationships at lower folate levels compared to other populations, in particular countries with folic acid fortification of foods [58, 59]. Finally, the multivariate statistical method used (i.e., Bayesian network learning) is more likely to capture complex interrelations between variables compared to the traditional use of univariate modeling of single components with post-analysis adjustment for multiple testing [29].

## Conclusion

In conclusion, we found no support for clear subtype-specific roles of one-carbon metabolism biomarkers and SNPs in CRC development, making differences in CRC molecular subtype distributions an unlikely explanation for the varying results on the role of one-carbon metabolism in CRC development across populations. A non-synonymous SNP in the *CTH* gene, rs1021737, was associated with an increased risk of *KRAS*-mutated CRC and decreased risk of *BRAF*-mutated CRC in the NSHDS, but the associations were not replicated in case-case analysis in the TCGA. Further investigation of the *CTH* gene in colorectal carcinogenesis with regards to *KRAS* and *BRAF* mutations or other molecular characteristics of the tumor may be warranted.

## Supporting information

S1 File Supplementary tables A-I.
(XLSX)

S2 File Supplementary figures A-B.
(DOCX)
