# Supplementary material for: One-carbon metabolism biomarkers and genetic variants in relation to colorectal cancer risk by KRAS and BRAF mutation status
Source: PLoS One. 2018 Apr 25;13(4):e0196233. doi: 10.1371/journal.pone.0196233 (PMC5919009; doi:10.1371/journal.pone.0196233)
Supplement: S2 File — Supplementary figures A-B. (DOCX) [file pone.0196233.s002.docx]

**Supplementary Figures**

**Myte R, Gylling B, Häggström J et al. One-carbon metabolism biomarkers and genetic variants in relation to colorectal cancer risk by *KRAS* and *BRAF* mutation status**

|  |
| --- |
| **Figure A. Plasma concentrations of metabolites in the transsulfuration pathway by genotypes of the *CTH* rs1021737 SNP.** P values were calculated using linear regression of log-transformed biomarker concentrations including the number of variant alleles of the SNPs as a continuous variable, adjusting for age, sex, cohort, sampling year, fasting status, and casecontrol status. The blue line represents the estimated regression line. |

|  |
| --- |
| **Figure B. Strength of association to CRC risk by (A) CIMP status (392 cases, 764 controls) and (B) MSI status (397 cases, 773 controls) for each biomarker and SNP**. Association strength is measured by edge confidence (%), defined as proportion of times an edge was present in 1000 bootstrap sample Bayesian networks calculated using the Hill-climbing algorithm. |
